# 1-(2-Chloro­phen­yl)-2-(isopropyl­amino)ethanol

**DOI:** 10.1107/S1600536809019953

**Published:** 2009-06-06

**Authors:** Zhan Tang, Min Xu, Gui-Ru Zheng, Hai Feng

**Affiliations:** a College of Pharmaceutical Sciences, Zhejiang University of Technology, Hangzhou 310014, People’s Republic of China; b Jinhua People’s Hospital, Jinhua 321000, People’s Republic of China

## Abstract

In the title compound, C_11_H_16_ClNO, the side chain of the ethyl­amine group is almost perpendicular to the benzene ring; the dihedral angle between the C/C/N plane of the ethyl­amine grouping and the benzene plane is 87.4 (2)°. An intramolecular N—H⋯O hydrogen bond occurs. In the crystal structure, mol­ecules are connected weakly by O—H⋯N hydrogen bonds, forming a tetra­mer around the 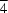 symmetry axis. The tetra­mers are linked weakly by a C—H⋯O hydrogen bond.

## Related literature

For a related structure, see: Koorts & Caira (1985 ▶). For the synthesis of the title compound, see; Koshinaka *et al.* (1978 ▶).
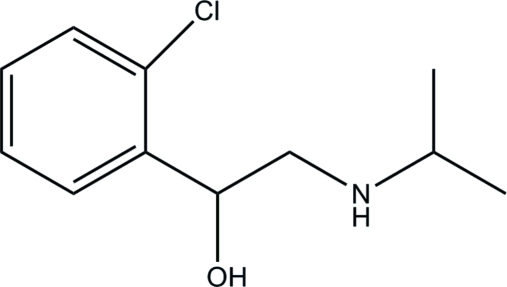


## Experimental

### 

#### Crystal data

C_11_H_16_ClNO
*M* *_r_* = 213.70
Tetragonal, 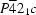

*a* = 14.0195 (5) Å
*c* = 12.1243 (4) Å
*V* = 2382.99 (14) Å^3^
*Z* = 8
Mo *K*α radiation
μ = 0.29 mm^−1^
*T* = 296 K
0.41 × 0.38 × 0.22 mm

#### Data collection

Rigaku R-AXIS RAPID diffractometer
Absorption correction: multi-scan (**ABSCOR**; Higashi, 1995 ▶) *T*_min_ = 0.871, *T*_max_ = 0.939
22063 measured reflections
2711 independent reflections
1796 reflections with *I* > 2σ(*I*)
*R*_int_ = 0.044

#### Refinement

*R*[*F* ^2^ > 2σ(*F* ^2^)] = 0.043
*wR*(*F* ^2^) = 0.098
*S* = 1.00
2711 reflections
131 parameters
H-atom parameters constrained
Δρ_max_ = 0.19 e Å^−3^
Δρ_min_ = −0.33 e Å^−3^
Absolute structure: Flack (1983 ▶), 1181 Friedel pairs
Flack parameter: 0.001 (1)

### 

Data collection: *PROCESS-AUTO* (Rigaku, 1998 ▶); cell refinement: *PROCESS-AUTO*; data reduction: *CrystalStructure* (Rigaku/MSC, 2004 ▶); program(s) used to solve structure: *SIR97* (Altomare *et al.*, 1999 ▶); program(s) used to refine structure: *SHELXL97* (Sheldrick, 2008 ▶); molecular graphics: *ORTEP-3* (Farrugia, 1997 ▶); software used to prepare material for publication: *SHELXL97*.

## Supplementary Material

Crystal structure: contains datablocks global, I. DOI: 10.1107/S1600536809019953/is2416sup1.cif

Structure factors: contains datablocks I. DOI: 10.1107/S1600536809019953/is2416Isup2.hkl

Additional supplementary materials:  crystallographic information; 3D view; checkCIF report

## Figures and Tables

**Table 1:** Hydrogen-bond geometry (Å, °)

*D*—H⋯*A*	*D*—H	H⋯*A*	*D*⋯*A*	*D*—H⋯*A*
O1—H201⋯N1^i^	0.82	1.97	2.765 (3)	164
N1—H301⋯O1	0.86	2.25	2.789 (3)	121
C6—H6⋯O1^ii^	0.93	2.45	3.283 (4)	149

Symmetry codes: (i) 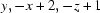; (ii) 
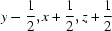
.

**Fig. 1.**
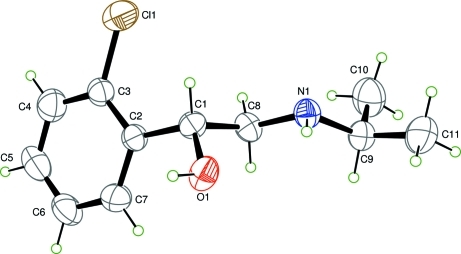
The molecular structure with atom labels, showing 40% probability displacement ellipsoids.

**Fig. 2.**
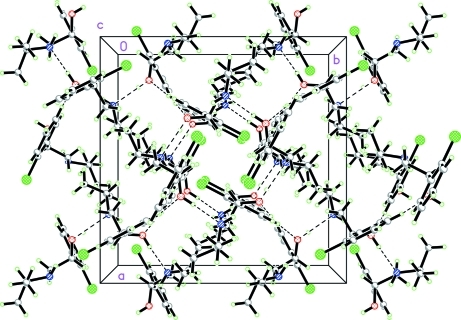
A packing diagram, viewed down along the c axis.

## supplementary crystallographic information

### Comment

The title compound (clorprenaline) is one of a series of structurally related β-adrenoceptorblocking drugs. Synthesis results have been reported in the literature (Koshinaka *et al.*, 1978). Clorprenaline was prepared by clorprenaline hydrochloride.

In the title compound (Fig. 1), there are no unusual bond distances or angles. The Cl atom and the phenyl plane is almost planar with the deviation of 0.0026 Å. The dihedral angle between the plane formed by C1 C2 C8 and the phenyl plane is 87.5°, which shows that the two planes are almost perpendicular. The C9—N1 distance of 1.473 (4) Å is shorter than the value of the similar bond distance of 1.502 Å (Koorts & Caira, 1985). The crystal structure indicates a possible intermolecular O—H···N interaction that might help to establish the crystal packing (Fig. 2).

### Experimental

Racemic clorprenaline hydrochloride (10 g, 0.047 mol), which was purchased from Hangzhou Chempro Tech Co., Inc. Hang Zhou, China, was dissolved in ethanol (100 ml) and NaOH (1.9 g, 0.047 mol) was dissolved in water (100 ml). The two solutions were mixed and the mixture was cooled for 3 h. The precipitate formed was filtered off, washed with water and dried. The crude product obtained was recrystallized from ethanol. Single crystals suitable for X-ray analysis were grown by slow evaporation at room temperature.

### Refinement

H atoms were placed in calculated positions and allowed to ride on their parent atoms with C—H = 0.93 (aromatic), 0.98 (methine), 0.97 (methylene), 0.96 (methyl), O—H = 0.82 and N—H = 0.858 Å, with *U*_iso_(H) = 1.2 or 1.5 times *U*_eq_ of the parent atoms.

### Crystal data

**Table:**

C_11_H_16_ClNO	*D*_x_ = 1.191 Mg m^−^^3^
*M**_r_* = 213.70	Mo *K*α radiation, λ = 0.71073 Å
Tetragonal, *P*42_1_*c*	Cell parameters from 13301 reflections
Hall symbol: P -4 2n	θ = 3.3–27.4°
*a* = 14.0195 (5) Å	µ = 0.29 mm^−^^1^
*c* = 12.1243 (4) Å	*T* = 296 K
*V* = 2382.99 (14) Å^3^	Block, colorless
*Z* = 8	0.41 × 0.38 × 0.22 mm
*F*(000) = 912	

### Data collection

**Table:**

Rigaku R-AXIS RAPID diffractometer	2711 independent reflections
Radiation source: RT	1796 reflections with *I* > 2σ(*I*)
graphite	*R*_int_ = 0.044
Detector resolution: 10.00 pixels mm^-1^	θ_max_ = 27.4°, θ_min_ = 3.3°
ω scans	*h* = −18→17
Absorption correction: multi-scan (*ABSCOR*; Higashi, 1995)	*k* = −18→18
*T*_min_ = 0.871, *T*_max_ = 0.939	*l* = −13→15
22063 measured reflections	

### Refinement

**Table:**

Refinement on *F*^2^	Hydrogen site location: inferred from neighbouring sites
Least-squares matrix: full	H-atom parameters constrained
*R*[*F*^2^ > 2σ(*F*^2^)] = 0.043	*w* = 1/[σ^2^(*F*_o_^2^) + (0.002*P*)^2^ + 1.96*P*] where *P* = (*F*_o_^2^ + 2*F*_c_^2^)/3
*wR*(*F*^2^) = 0.098	(Δ/σ)_max_ < 0.001
*S* = 1.00	Δρ_max_ = 0.19 e Å^−^^3^
2711 reflections	Δρ_min_ = −0.33 e Å^−^^3^
131 parameters	Extinction correction: *SHELXL97* (Sheldrick, 2008), Fc^*^=kFc[1+0.001xFc^2^λ^3^/sin(2θ)]^-1/4^
0 restraints	Extinction coefficient: 0.0308 (10)
Primary atom site location: structure-invariant direct methods	Absolute structure: Flack (1983), 1181 Friedel pairs
Secondary atom site location: difference Fourier map	Flack parameter: 0.001 (1)

### Special details

**Table:**

Geometry. All e.s.d.'s (except the e.s.d. in the dihedral angle between two l.s. planes) are estimated using the full covariance matrix. The cell e.s.d.'s are taken into account individually in the estimation of e.s.d.'s in distances, angles and torsion angles; correlations between e.s.d.'s in cell parameters are only used when they are defined by crystal symmetry. An approximate (isotropic) treatment of cell e.s.d.'s is used for estimating e.s.d.'s involving l.s. planes.
Refinement. Refinement of *F*^2^ against ALL reflections. The weighted *R*-factor *wR* and goodness of fit *S* are based on *F*^2^, conventional *R*-factors *R* are based on *F*, with *F* set to zero for negative *F*^2^. The threshold expression of *F*^2^ > σ(*F*^2^) is used only for calculating *R*-factors(gt) *etc*. and is not relevant to the choice of reflections for refinement. *R*-factors based on *F*^2^ are statistically about twice as large as those based on *F*, and *R*- factors based on ALL data will be even larger.

### Fractional atomic coordinates and isotropic or equivalent isotropic displacement parameters (Å^2^)

**Table:**

	*x*	*y*	*z*	*U*_iso_*/*U*_eq_	
Cl1	0.89802 (8)	0.90968 (7)	0.76779 (8)	0.0866 (3)	
O1	0.82940 (16)	1.14673 (15)	0.54268 (18)	0.0616 (6)	
H201	0.8801	1.1727	0.5581	0.074*	
C2	0.81036 (19)	1.07851 (19)	0.7258 (2)	0.0484 (6)	
C1	0.8209 (2)	1.06095 (19)	0.6030 (2)	0.0492 (7)	
H1	0.8769	1.0208	0.5896	0.059*	
N1	0.74199 (17)	0.99152 (18)	0.4402 (2)	0.0527 (6)	
H301	0.7679	1.0439	0.4192	0.063*	
C4	0.8307 (3)	1.0339 (3)	0.9173 (3)	0.0717 (10)	
H4	0.8532	0.9910	0.9697	0.086*	
C3	0.8417 (2)	1.0153 (2)	0.8060 (2)	0.0557 (7)	
C8	0.7325 (2)	1.0115 (2)	0.5591 (2)	0.0538 (7)	
H8A	0.7227	0.9521	0.5987	0.065*	
H8B	0.6772	1.0517	0.5714	0.065*	
C9	0.6502 (2)	0.9762 (2)	0.3839 (3)	0.0658 (9)	
H9	0.6062	1.0272	0.4054	0.079*	
C7	0.7657 (2)	1.1617 (2)	0.7621 (3)	0.0649 (8)	
H7	0.7436	1.2054	0.7104	0.078*	
C11	0.6655 (3)	0.9809 (3)	0.2604 (3)	0.0907 (12)	
H11A	0.7094	0.9319	0.2385	0.109*	
H11B	0.6911	1.0422	0.2411	0.109*	
H11C	0.6057	0.9717	0.2233	0.109*	
C6	0.7534 (2)	1.1809 (3)	0.8735 (3)	0.0753 (10)	
H6	0.7233	1.2367	0.8959	0.090*	
C10	0.6073 (3)	0.8813 (3)	0.4176 (3)	0.0885 (12)	
H10A	0.5965	0.8810	0.4958	0.106*	
H10B	0.6505	0.8307	0.3986	0.106*	
H10C	0.5479	0.8721	0.3798	0.106*	
C5	0.7860 (3)	1.1172 (3)	0.9497 (3)	0.0779 (11)	
H5	0.7781	1.1300	1.0244	0.093*	

### Atomic displacement parameters (Å^2^)

**Table:**

	*U*^11^	*U*^22^	*U*^33^	*U*^12^	*U*^13^	*U*^23^
Cl1	0.1254 (9)	0.0656 (5)	0.0688 (5)	0.0248 (5)	−0.0095 (6)	0.0035 (5)
O1	0.0652 (14)	0.0607 (13)	0.0588 (12)	−0.0115 (10)	−0.0115 (11)	0.0144 (10)
C2	0.0470 (14)	0.0513 (15)	0.0470 (14)	−0.0033 (12)	0.0018 (13)	−0.0009 (13)
C1	0.0505 (16)	0.0506 (16)	0.0466 (15)	0.0013 (13)	−0.0003 (13)	0.0046 (12)
N1	0.0532 (14)	0.0547 (14)	0.0501 (13)	−0.0047 (12)	−0.0052 (11)	−0.0027 (12)
C4	0.086 (3)	0.082 (3)	0.0473 (18)	−0.006 (2)	−0.0079 (17)	−0.0014 (16)
C3	0.0620 (18)	0.0542 (17)	0.0509 (16)	−0.0040 (15)	−0.0033 (14)	0.0002 (13)
C8	0.0536 (17)	0.0556 (17)	0.0522 (16)	−0.0052 (14)	0.0036 (14)	−0.0057 (14)
C9	0.0605 (19)	0.062 (2)	0.075 (2)	0.0040 (16)	−0.0155 (17)	−0.0085 (16)
C7	0.0632 (19)	0.0651 (19)	0.066 (2)	0.0135 (15)	0.0028 (17)	−0.0066 (17)
C11	0.106 (3)	0.092 (3)	0.074 (2)	0.004 (2)	−0.032 (2)	−0.007 (2)
C6	0.065 (2)	0.086 (3)	0.075 (2)	0.0079 (19)	0.0035 (19)	−0.025 (2)
C10	0.070 (2)	0.085 (3)	0.110 (3)	−0.021 (2)	−0.011 (2)	−0.007 (2)
C5	0.075 (2)	0.104 (3)	0.0548 (19)	−0.012 (2)	0.0057 (18)	−0.025 (2)

### Geometric parameters (Å, °)

**Table:**

Cl1—C3	1.741 (3)	C8—H8B	0.9700
O1—C1	1.413 (3)	C9—C10	1.516 (5)
O1—H201	0.8200	C9—C11	1.514 (5)
C2—C3	1.387 (4)	C9—H9	0.9800
C2—C7	1.395 (4)	C7—C6	1.387 (5)
C2—C1	1.516 (4)	C7—H7	0.9300
C1—C8	1.517 (4)	C11—H11A	0.9600
C1—H1	0.9800	C11—H11B	0.9600
N1—C9	1.473 (4)	C11—H11C	0.9600
N1—C8	1.475 (4)	C6—C5	1.364 (5)
N1—H301	0.8580	C6—H6	0.9300
C4—C5	1.382 (5)	C10—H10A	0.9600
C4—C3	1.383 (4)	C10—H10B	0.9600
C4—H4	0.9300	C10—H10C	0.9600
C8—H8A	0.9700	C5—H5	0.9300
			
C1—O1—H201	109.5	N1—C9—C11	109.2 (3)
C3—C2—C7	117.1 (3)	C10—C9—C11	111.2 (3)
C3—C2—C1	123.6 (3)	N1—C9—H9	108.7
C7—C2—C1	119.3 (3)	C10—C9—H9	108.7
O1—C1—C8	106.1 (2)	C11—C9—H9	108.7
O1—C1—C2	112.2 (2)	C6—C7—C2	121.7 (3)
C8—C1—C2	109.8 (2)	C6—C7—H7	119.2
O1—C1—H1	109.6	C2—C7—H7	119.2
C8—C1—H1	109.6	C9—C11—H11A	109.5
C2—C1—H1	109.6	C9—C11—H11B	109.5
C9—N1—C8	113.7 (2)	H11A—C11—H11B	109.5
C9—N1—H301	110.9	C9—C11—H11C	109.5
C8—N1—H301	99.5	H11A—C11—H11C	109.5
C5—C4—C3	119.2 (3)	H11B—C11—H11C	109.5
C5—C4—H4	120.4	C5—C6—C7	119.4 (3)
C3—C4—H4	120.4	C5—C6—H6	120.3
C4—C3—C2	121.9 (3)	C7—C6—H6	120.3
C4—C3—Cl1	118.1 (3)	C9—C10—H10A	109.5
C2—C3—Cl1	120.0 (2)	C9—C10—H10B	109.5
N1—C8—C1	110.9 (2)	H10A—C10—H10B	109.5
N1—C8—H8A	109.5	C9—C10—H10C	109.5
C1—C8—H8A	109.5	H10A—C10—H10C	109.5
N1—C8—H8B	109.5	H10B—C10—H10C	109.5
C1—C8—H8B	109.5	C6—C5—C4	120.8 (3)
H8A—C8—H8B	108.1	C6—C5—H5	119.6
N1—C9—C10	110.5 (3)	C4—C5—H5	119.6
			
C3—C2—C1—O1	−150.5 (3)	C9—N1—C8—C1	−159.3 (3)
C7—C2—C1—O1	30.5 (4)	O1—C1—C8—N1	59.9 (3)
C3—C2—C1—C8	91.8 (3)	C2—C1—C8—N1	−178.6 (2)
C7—C2—C1—C8	−87.2 (3)	C8—N1—C9—C10	−71.4 (4)
C5—C4—C3—C2	1.0 (5)	C8—N1—C9—C11	166.0 (3)
C5—C4—C3—Cl1	−179.8 (3)	C3—C2—C7—C6	0.3 (5)
C7—C2—C3—C4	−0.9 (5)	C1—C2—C7—C6	179.4 (3)
C1—C2—C3—C4	−180.0 (3)	C2—C7—C6—C5	0.2 (5)
C7—C2—C3—Cl1	179.9 (2)	C7—C6—C5—C4	−0.2 (6)
C1—C2—C3—Cl1	0.9 (4)	C3—C4—C5—C6	−0.4 (6)

### Hydrogen-bond geometry (Å, °)

**Table:**

*D*—H···*A*	*D*—H	H···*A*	*D*···*A*	*D*—H···*A*
O1—H201···N1^i^	0.82	1.97	2.765 (3)	164
N1—H301···O1	0.86	2.25	2.789 (3)	121
C6—H6···O1^ii^	0.93	2.45	3.283 (4)	149

Symmetry codes: (i) *y*, −*x*+2, −*z*+1; (ii) *y*−1/2, *x*+1/2, *z*+1/2.
